# RhoGTPase stimulation is associated with strontium chloride treatment to counter simulated microgravity-induced changes in multipotent cell commitment

**DOI:** 10.1038/s41526-016-0004-6

**Published:** 2017-01-25

**Authors:** Fiona Louis, Wafa Bouleftour, Aline Rattner, Marie-Thérèse Linossier, Laurence Vico, Alain Guignandon

**Affiliations:** 1 INSERM, U1059, SAINBIOSE, Saint-Etienne, F-42023 France; 2 Université de Lyon, Saint-Etienne, F-42023 France

## Abstract

Microgravity-related cytoskeletal disorganization is associated with an altered balance between osteoblastogenesis and adipogenesis of multipotent cells. Strontium chloride is known to increase osteoblastogenesis and repress adipogenesis, but its effects in microgravity-related conditions have not been established. Our goal was to investigate early events in this process, focusing on RhoGTPases as controllers of cytoskeletal organization leading to stem cell commitment. We cultivated C3H10T1/2 on microspheres using a rotating wall vessel bioreactor (NASA) in order to simulate microgravity-related conditions in adipogenesis and osteoblastogenesis conditions independently. We observed that rotating wall vessel cultures presented increased adipogenesis, while osteoblastogenesis was reduced. Strontium-treated multipotent cells presented a significant repression in adipogenesis (−90 %, *p* < 0.001 PPARyD8) and an activation of osteoblastogenesis (+95 %, *p* < 0.001 bone sialoprotein and osteopontin D8), even in gravity altered conditions. We established that concomitant RhoA/Rac1 activations were associated with osteoblastogenesis enhancement and adipogenesis limitation in uncommitted cells. As vascular endothelial growth factor splicing is mechanosensitive and its signaling is central to stem cell commitment, we investigated vascular endothelial growth factor production, isoforms and receptors expressions in our conditions. We observed that vascular endothelial growth factor and receptors expressions were not significantly affected, but we found that presence of soluble vascular endothelial growth factor was associated with RhoA/Rac1 activations, whereas sequestration of vascular endothelial growth factor by cells was associated with RhoA/Rac1 inhibitions. We propose that strontium triggers secretion of vascular endothelial growth factor and the subsequent Rac1 and RhoA activations leading to repression of adipogenesis and osteogenesis stimulation validating strontium as a counter measure for microgravity-induced alteration of cell commitment.

## Countering Bone Loss in Space

A chemical element naturally found for instance in seafood or grains, could counter bone loss from long-term spaceflight. Alain Guignandon and colleagues from the Université de Lyon à St-Etienne in France exposed multipotent embryonic fibroblasts to microgravity conditions similar to those found in space. They found the balance shifted in these stem cells from differentiating to bone-forming cells (osteoblasts) to differentiating to fatty-tissue forming cells (adipocytes). When the cells were treated with strontium, the shift toward osteoblastogenesis was regained. Strontium achieves this by sustaining the activity of two proteins that play a role in bone development but are suppressed in space. Strontium’s effect on the proteins could happen via release of vascular endothelial growth factor, which, under normal gravity conditions, plays a role in committing the cell to differentiation into osteoblasts rather than adipoyctes.

## Introduction

Bone loss is currently one of the most serious health hazards of long-term spaceflight. Continuous and progressive loss of calcium in weight-bearing bones is observed in both humans and animals during exposure to microgravity,^1^ which is thought to be due to the loss of mechanical stress and duration of flights.^2–4^ In microgravity, the balance between osteoblastogenesis (OB) and adipogenesis (AD) for bone marrow mesenchymal stem cells (MSC) shifts in favor of adipocytes,^5^ resulting in reduced bone mass and an altered bone architecture.^6^ Moreover, osteoporotic bones display an increased accumulation of adipocytes in the trabecular bone marrow space.^7–9^

Adipocytes and osteoblasts differentiate from a common mesenchymal precursor cell. This raises the question of whether bone loss is a consequence of gravity alterations affecting osteoblast/adipocyte fates from MSC. Our understanding of the control of this fate is incomplete. We suggest that key regulators of multipotent cells commitment such as RhoGTPases are implicated. Small GTP-binding proteins of the Rho family (RhoA, Cdc42, Rac1) are key regulators for the organization and turnover of the cytoskeleton, formation of cell–matrix adhesions, transcriptional control of gene expression, cell survival and proliferation. In particular, RhoA, which is characterized for its role in stress fiber formation in cells,^10^ has been shown to be implicated in MSC lineage commitment.^11,12^ It is, therefore, likely that Rho-GTPases are directly involved in cellular gravity perception and may participate in the alterations induced in the absence of gravity,^13–15^ since the overexpression of constitutively active RhoA in cells exposed to microgravity conditions leads to a recovery of stress fibers comparable with that of cells cultured in normal gravity.^16^ Concerning Rac1, its signaling in bone marrow stromal cells showed that it is required for normal bone development^17^ and regulates shear stress-driven β-catenin signaling in osteoblasts.^18^ So reduced activations of both RhoA and Rac1 during space flight may contribute to the altered MSC differentiation observed.^5,15,19^ However, the pathway by which RhoA or Rac1 activities regulates the differentiation of MSC has not yet been elucidated.

We propose to test the role of vascular endothelial growth factor A (VEGF) in the control of our multipotent cells model for the followings reasons. According to the results of Liu et al*.*,^20^ VEGF signaling controls multipotent cell commitment and other studies even suggest that VEGF stimulates osteoblast differentiation.^21–23^ In detail, when VEGF linked to its receptor 2 (Flk-1) translocates to the nucleus, the cell is committed to osteogenesis (OB) by way of a LaminA/C-dependent regulation of Runx2 (OB master gene) transcription. If VEGF/Flk-1 is unable to translocate to the nucleus, Runx2 transcription is repressed and AD is activated. Furthermore, VEGF alternative splicing is known to be regulated by mechanical stresses such as stretching/compression^24–26^ or microgravity.^27^ Finally, matrix-bound or soluble VEGF possesses distinct signaling abilities^28,29^ that might be linked to its differential ability to stimulate RhoGTPases.

In this context, our first objective was to evaluate the ability of multipotent cells to differentiate into committed osteoprogenitor or pre-adipocytes under conditions of simulated microgravity and to analyze the association between commitment and levels of RhoA and Rac1 activities as well as VEGF expression in both lineages. Strontium has been shown to promote OB^30^ and prevent AD of MSC.^31^ Thus, our second objective was to establish in our cell model the role of strontium in the activation of RhoGTPases and VEGF pathways.

We cultivated multipotent embryonic fibroblasts (C3H10T1/2) on microspheres in the NASA-approved rotating wall vessel (RWV) in order to simulate microgravity-related conditions. The RWV model had been widely used in simulated microgravity condition experiments and is considered to be a spaceflight analogue allowing reconstitution of gravity alterations on RhoGTPases^15,19^and gene/protein regulations.^5,19,32,33^ This is a convenient model to predict reduced gravity results without delays associated with true spaceflight experiments. The C3H10T1/2 cell line was chosen for its known ability to permit commitments of both adipocytes and osteoblasts from the same cells and equal duration treatments times, allowing direct comparisons of these two pathways.^34,35^These cells also provided the large proliferation rate needed to obtain the 20.10^6^ cells required per experiment in the bioreactor, in a context of avoiding animal experimentation. Cells were grown under conditions where both AD (collagen plastic beads) and osteogenesis (OB; apatite coated on collagen plastic beads) were promoted in static conditions (three-dimensional (3D) controls). These 3D cultures were either treated or not with 5 mM of strontium for up to 8 days of differentiation. We evaluated RhoA and Rac1 activities during AD and OB as well as VEGF isoforms (120, 164 and 188), in addition to its receptors expressions. We established that strontium was able to sustain RhoA and Rac1 activities under RWV conditions and that this dual activation was associated with expression and release of VEGF. We propose that strontium treatment of uncommitted cells triggers VEGF secretion and RhoA/Rac1 activation, leading to OB promotion and AD restriction to finally help bone cells to resist against microgravity conditions.

## Results

### Culture in RWV conditions affects MSC differentiation

**Fig. 1:**
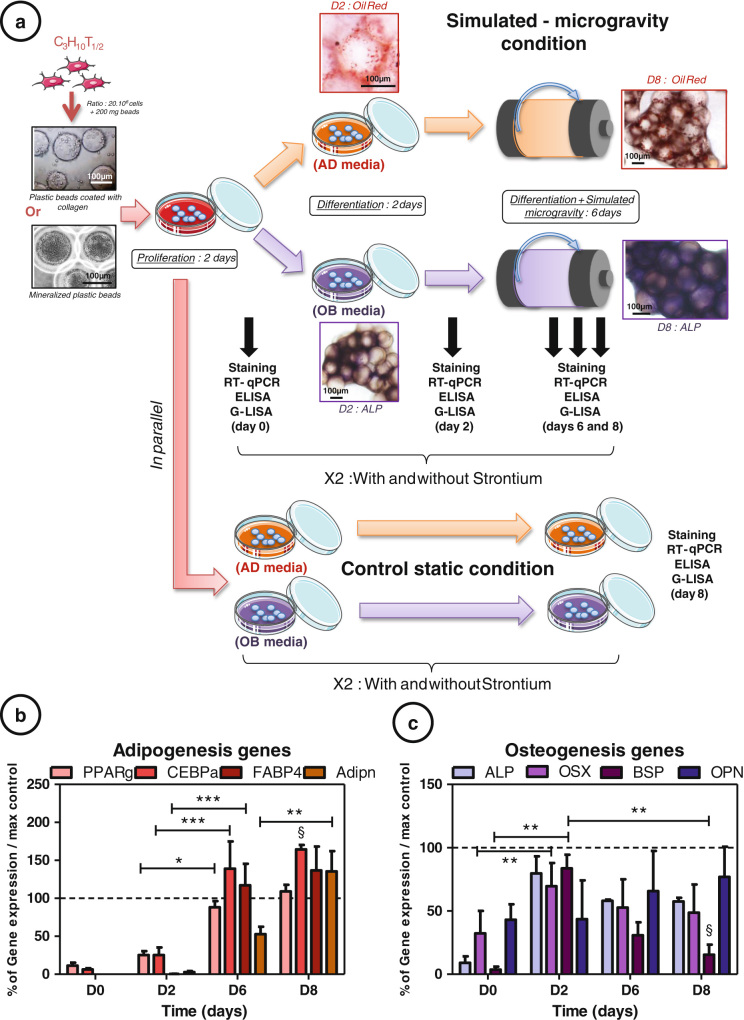
Culture in rotating wall vessel conditions affects mesenchymal stem cell differentiation: **a** C3H10T1/2 cells were cultured 2 days on plastic beads or mineralized plastic beads in αMEM media in order to promote cell proliferation. They were then induced 2 days in differentiation media (for adipogenesis (AD) orosteogenesis (OB) with or without 5 mM strontium) before being cultured in simulated-microgravity condition or kept in static control condition in plastic dishes. Extraction of aggregates were realized in order to perform Oil *Red* O staining (**b**), ALP staining (**c**), RT-quantitative PCR, ELISA and G-Lisa at days 0, 2, 6 and 8 for rotating condition and at days 0, 2 and 8 for static control condition. Each condition was repeated three times independently. Adipogenic [**b**: peroxisome proliferator-activated receptor γ (PPARγ), CCAAT-enhancer-binding proteins α (C/EBPα), fatty acid-binding protein (FABP4) and adiponectin] and osteogenic [**c**: alkaline phosphatase (ALP), osterix (OSX), bone sialoprotein (BSP) and osteopontin (OPN)] gene expressions were measured by RT-quantitative PCR throughout the culture in rotating condition at days 0, 2, 6 and 8. The data were normalized by the gene expressions obtained in the control static condition at day 8. In all, 100 % corresponds to the maximal control differentiation rate reached in control. Error *bars* represent S.E.M. *p < 0.05, **p < 0.01 and ***p < 0.001 compared to media without strontium, or compared to conditions indicated by the horizontal *bars* (*N* = 3 independent experiments). *Section* symbol represents statistical comparison between rotating and static condition with *p* < 0.05

MSC C3H10T1/2 were seeded onto nonmineralized or mineralized beads. They grew in static condition during 4 days and then in simulated microgravity, or were kept in static condition for up to 8 days (Fig. 1a). AD markers (peroxisome proliferator-activated receptor γ2 (PPARγ), CCAAT-enhancer-binding proteins α (C/EBPα), fatty acid-binding protein (FABP4) and adiponectin) and osteogenesis markers (alkaline phosphatase (ALP), osterix (OSX), bone sialoprotein (BSP) and osteopontin (OPN)) gene expressions, normalized by the static control at day 8, allowed to assess the impact of the rotation on the MSC commitment (Figs. 1b, c). C/EBPα and FABP4 showed a higher expression from day 6 (+64 % at D8 for C/EBPα, *p* < 0.05) and PPARγ and adiponectin from day 8, compared to control conditions. Osteogenic genes stayed below the control expression throughout the culture (−77 % at D8 for BSP, *p* < 0.05). The four adipogenic genes significantly increased during the rotating culture (up to +130 % for FABP4 between D0 and D8, *p* < 0.001) and osteogenic genes were reduced (up to −68 % between D2 and D8, *p* < 0.01 for BSP). These results validated the spaceflight analogue effect of the RWV culture; i.e., inhibition or delay of osteogenic commitment and induction or acceleration of the adipogenic one.

### Strontium acts as a negative regulator of AD and a positive regulator of OB

**Fig. 2:**
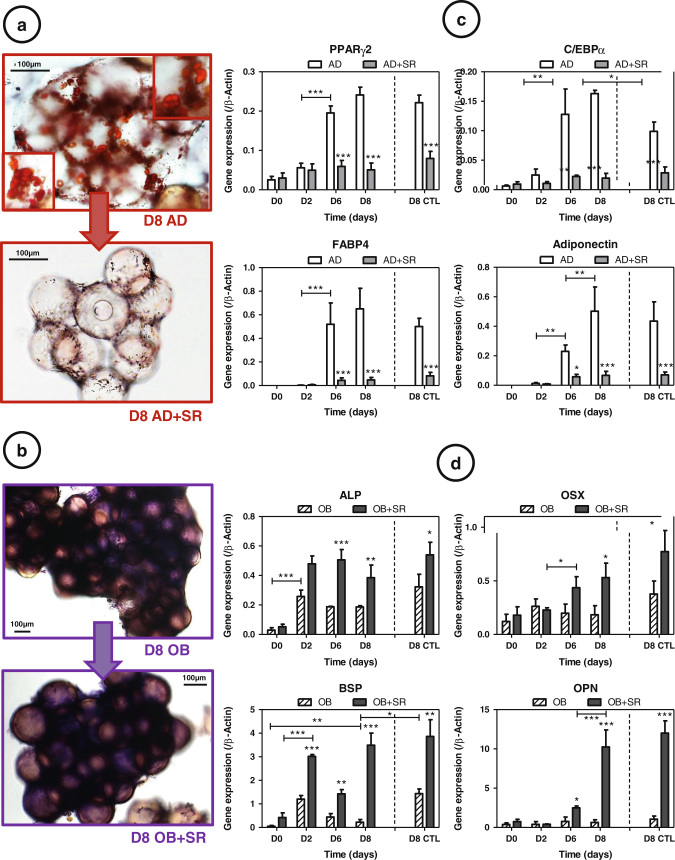
Srontium (SR) acts as a negative regulator of adipogenesis (AD) and a positive regulator of osteogenesis (OB): **a** Oil Red staining in AD conditions. **b** Alkaline phosphatase staining in OB conditions, **c** Adipogenesis markers were measured by RT-quantitative PCR during the culture [peroxisome proliferator-activated receptor γ (PPARγ), CCAAT-enhancer-binding proteins α (C/EBPα), fatty acid-binding protein (FABP4) and adiponectin], and **d** osteogenesis markers [alkaline phosphatase (ALP), osterix (OSX), bone sialoprotein (BSP) and osteopontin (OPN)], in AD or OB media, with or without 5 mM strontium. Day 8 of control static condition corresponds to the D8 CTL data. Error bars represent S.E.M. *p < 0.05, **p < 0.01 and ***p < 0.001 compared to media without strontium or compared with conditions indicated by the horizontal *bars* (*N* = 3 independent experiments)

The effect of strontium on MSC commitment was appraised by staining observations (Figs. 2a, b). As shown, the proportion of cells showing positive staining for Oil Red O, and thus intracellular lipid accumulation, was lower in strontium-treated cultures as compared to untreated cells (Fig. 2a). Similarly, concerning alkaline phosphatase staining, cells in both groups were stained very positively and showed no differences (Fig. 2b). To assess how strontium treatment affects differentiating adipocytes, real-time polymerase chain reaction (RT-PCR) results were analyzed. Under rotating conditions we observed that AD markers increased continually during the culture (Fig. 2c) (up to+130 %, *p* < 0.001 for FABP4 between D0–D8); in the same time, OB gene expression did not increase during the 8 days of culture and even sometimes significantly decreased (BSP, −68 %, *p* < 0.01) (Fig. 2d). RWV significantly promoted AD (*p* < 0.05 for C/EBPα), without allowing osteogenic induction (*p* < 0.05 for BSP). As expected^31^ strontium dramatically inhibited AD (down to −90 %, *p* < 0.001 for FABP4 at day 8) and also promoted OB (until +95 %, *p* < 0.001 for BSP and OPN at day 8) (Fig. 2c, d)^30^ when used at a concentration of 5 mM.

### Strontium regulates RhoA and Rac-1 activities in uncommitted adipocytes and osteoblasts

**Fig. 3:**
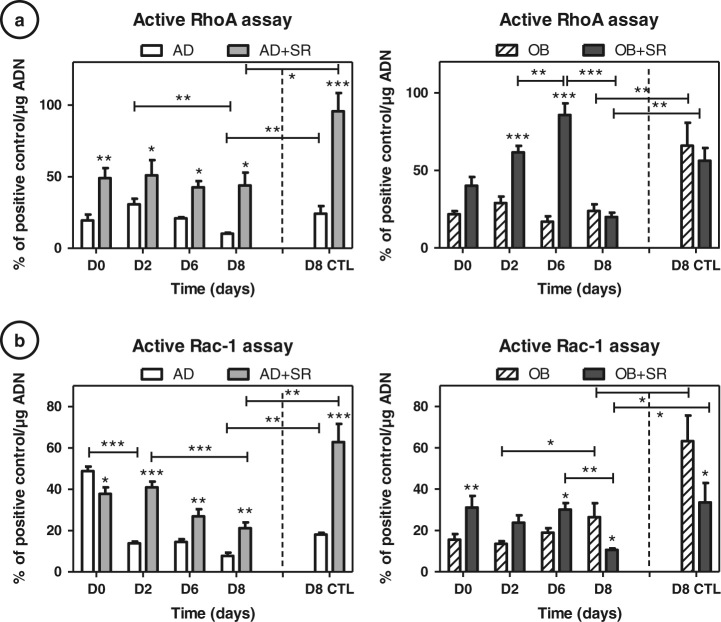
Strontium (SR) regulates RhoA and Rac-1 activities in commited adipocytes and osteoblasts: C3H10T1/2 cells were cultured 2, 6 and 8 days in adipogenesis (AD) or osteogenesis (OB) media and simulated-microgravity, with or without 5 mM strontium. Cells were then harvested for G-LISA assays of RhoA (**a**) and Rac1 (**b**). Day 8 of control static condition corresponds to the D8 CTL data. Positive controls for RhoA and Rac1 activities are provided in the assay kit, activities of RhoGTpases data were normalized by this positive control. Error *bars* represent S.E.M. *p < 0.05, **p < 0.01 and ***p < 0.001 compared to media without strontium, or compared to conditions indicated by the horizontal *bars* (*N* = 4independant experiments)

As GTPases have been implicated in multipotent cell commitment, we used specific G-LISA to investigate activities of RhoA- and Rac1-GTP under both OB and AD conditions of culture, with or without strontium treatment (Fig. 3), compared to day 8 static control condition (D8 CTL). Active RhoA decreased during rotating AD from day 2 to day 8 (−65 %, *p* < 0.01), whereas it was maintained in intermediate amounts during rotating osteogenesis (around 20 %) (Fig. 3a). Rac-1 tends to increase during osteogenesis(+40 %, *p* < 0.05 between D2–D8) and decrease during AD, mostly at the beginning (−64 %, *p* < 0.001) in the RWV (Fig. 3b). Both RhoA and Rac1 were shown to be significantly inhibited in an altered gravity condition, compared to the static control condition (−58 % for RhoA AD, −64 % for RhoA OB, −57 % for Rac1 AD and −58 % for Rac1 OB, *p* < 0.01). According to the differentiation stage, we could clearly identify two types of strontium responses (day 2: majority of uncommitted cells and day 8: majority of just committed cells). After 4 days of induction, regardless of the conditions, we observed significant increases by strontium in RhoA-GTP and Rac1-GTP activities (e.g., for RhoA: +52 %, *p* < 0.01 at D6 in AD and +75 % at D6 in OB, *p* < 0.001; for Rac 1: +43 %, *p* < 0.01 at D6 in AD and +34 %, *p* < 0.05 at D6 in OB). A similar profile was found up to 6 days of culture under both AD and OB conditions. Concerning RhoGTPases activities at day 8, at which time a majority of cells has been committed (according to the molecular profile), we found that RhoA was no more activated by the strontium treatment than untreated OB cells. On the contrary, RhoA activity was still significantly increased under AD conditions under strontium (+65 %, *p* < 0.05 D8 vs. untreated matching samples), which correspond in this condition to still uncommitted cells.

Similarly to RhoA, we found at day 8 that Rac1 activity became particularly decreased under strontium as compared to untreated cells in OB conditions (−62 %, *p* < 0.05) and that Rac1 activity was significantly increased under strontium and AD conditions (+49 %, *p* < 0.01). The dual strontium-induced RhoGTPases were found in a similar way for the day 8 static control conditions. Only adipocyte commitment showed a higher induction through strontium action (+72 %, *p* < 0.001 for RhoA and +71 %, *p* < 0.001 for Rac1). These results raise the question of a differential RhoGTPases activation attributed to a differential VEGFR signaling. RhoGTPases signaling could be altered by the quantity of VEGF protein available, differential expressions of VEGF receptors or VEGF isoforms.

### Differential regulation of cell-associated and soluble VEGF production by strontium

**Fig. 4:**
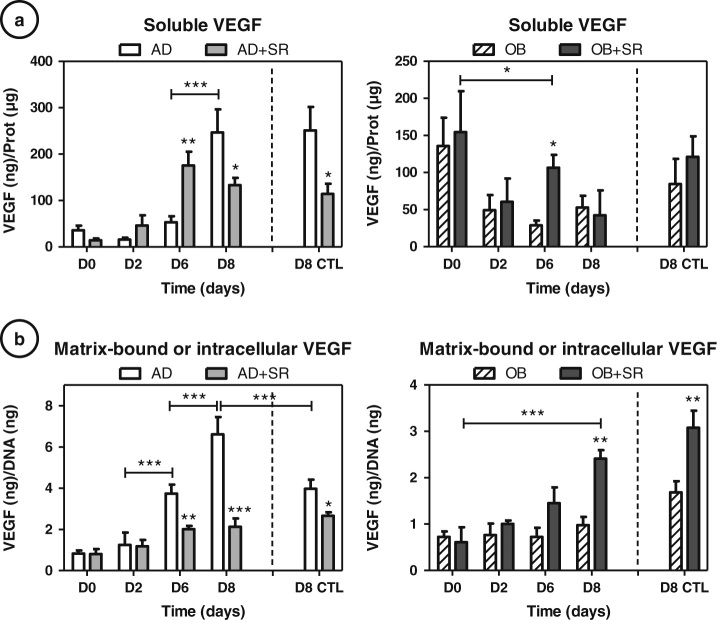
Differential regulation of cell-associated and soluble VEGF by strontium: ELISA of VEGF protein was performed at days 2, 6 and 8 on culture supernatants to quantify soluble VEGF proteins **a**, normalized by total DNA as well as in cell/matrix lysates to quantify matrix-bound or cell-associated VEGF **b**, normalized by total proteins. Day 8 of control static condition corresponds to the D8 CTL data. Error *bars* represent S.E.M. *p < 0.05, **p < 0.01 and ***p < 0.001 compared to media without strontium, or compared to conditions indicated by the horizontal *bars* (*N* = 3 independent experiments). AD: adipogenic, OB: osteogenic, SR: strontium

VEGF seems to play important regulatory roles on the control of osteoblast/adipocyte fates in MSC.^20^ Enzyme-linked immunosorbent assay (ELISA) of VEGF protein was thus performed on culture supernatants to quantify the soluble VEGF (VEGFs) proteins (Fig. 4a) as well as in cell/matrix lysates to quantify cell-associated VEGF (VEGFc) (Fig. 4b).VEGFc comprised matrix-bound VEGF and intracellular VEGF.

During AD, both soluble and cell-associated VEGF increased from day 2 to day 8 of culture. The effects of strontium on VEGF under AD condition showed an inhibition of VEGFc throughout the culture and seemed more complex for VEGFs because its secretion initially increased (day 2 and 6) and decreased at day 8. Similarly, at day 8, the strontium treatment significantly reduced both the content of VEGFs and VEGFc (−41 %, *p* < 0.05 and −65 %, *p* < 0.001, respectively) (Fig. 4a, b). Similar expressions and inhibition rates were observed for the static control, except for AD which was significantly found lower for VEGFc (−40 % compared to control, *p* < 0.001).

Concerning osteoblastic conditions, VEGFs and VEGFc production remained constant during all the duration of the experiment, which might be coherent with the limitation of osteogenesis in these rotated cultures. When strontium was added to the culture, we observed a significant increase in VEGFc secretion only at day 8 (+54 %, *p* < 0.01) and no other notable changes were found. For VEGFs production, we observed that strontium increased production at day 6 (+78 %, *p* < 0.05), matching with the beginning of OB commitment as seen from the molecular profile (Fig. 2). 3D static controls showed identical effects of strontium demonstrating that strontium effects were not dependent on the mechanical status of the culture.

In summary, we found that strontium inhibited VEGFs and VEGFc production in committed adipocytes and promoted only VEGFc in committed osteoblasts in RWV cultures as well as in 3D static control cultures.

### Strontium downregulates Nrp-1 receptor expression

**Fig. 5:**
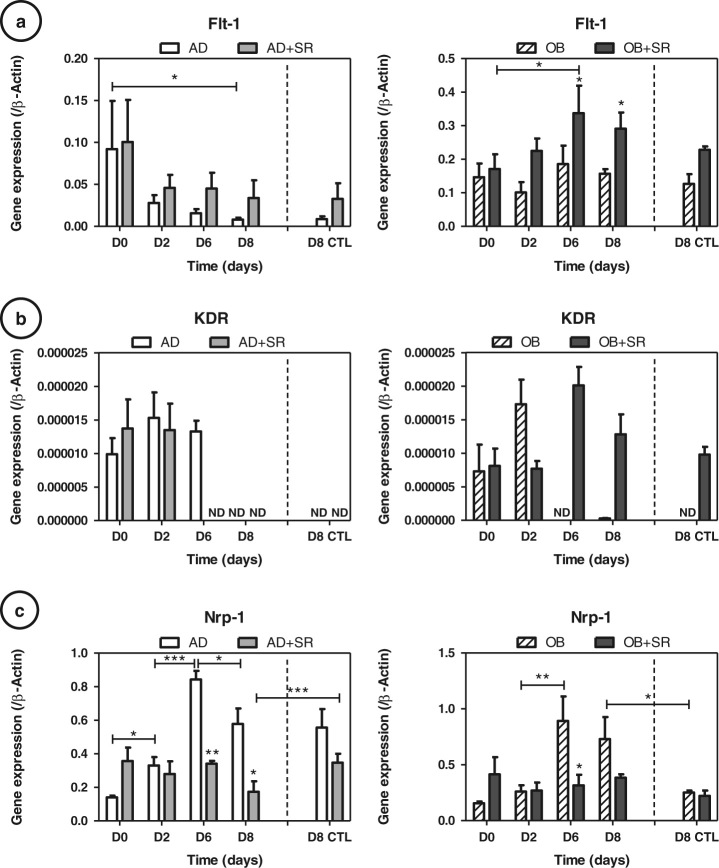
Strontium (SR) downregulates Nrp-1 receptor expression: **a** VEGF receptor 1 (Flt-1), **b** VEGF receptor 2 (KDR) and **c** neuropilin 1 (Nrp-1) gene expressions were measured at days 2, 6 and 8 by RT-quantitative PCR during the cultures in adipogenic (AD) or osteogenic (OB) media, with or without 5 mM strontium. Day 8 of control static condition corresponds to the D8 CTL data. Error bars represent S.E.M. *p <  0.05, **p < 0.01 and ***p < 0.001 compared to media without strontium or compared with conditions indicated by the horizontal bars (*N* = 3 independent experiments)

Paracrine VEGF signaling is mediated by the tyrosine kinase receptors VEGFR1 (Flt-1), VEGFR2 (KDR/Flk-1) and Nrp-1 (Fig. 5). KDR expression was dramatically reduced as compared to Flt-1 or Nrp-1, making difficult any interpretation concerning strontium effects. Strontium showed activation of Flt-1 in both adipocytes and osteoblasts (up to +65 % for AD and +45 %, *p* < 0.05 for OB at day 8). Of note, the Nrp-1 profile was identical in adipogenic and osteogenic conditions. Nrp-1 expressions were significantly decreased with strontium treatment of uncommitted or differentiated cells (up to −56 %, *p* < 0.05 for AD at day 6; Fig. 5c). Static controls were similar to the rotating conditions at day 8; only Nrp-1 was found significantly reduced in controls (−52 %, *p* < 0.05), dampening the strontium effects.

### Strontium differentially regulates VEGF188 isoform expression in OB and AD conditions

**Fig. 6:**
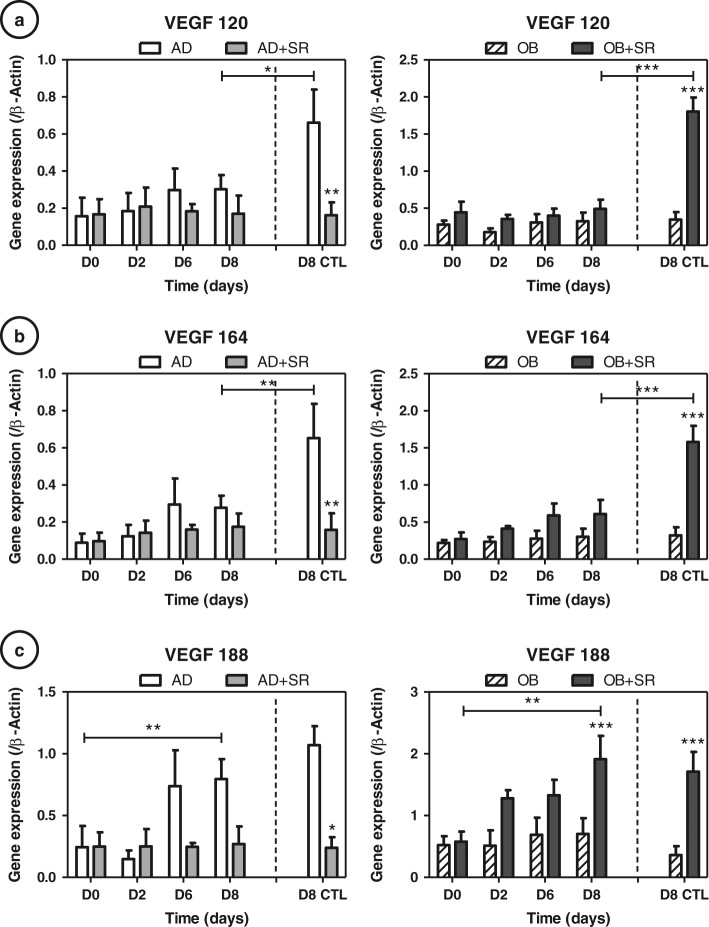
Strontium (SR) differentially regulates VEGF188 isoform expression in osteogenic (OB) and adipogenic(AD) conditions: VEGF isoforms (**a**: 120, **b**: 164 and **c**: 188) were measured at days 2, 6 and 8 by RT-quantitative PCR during the cultures in AD or OB media, with or without 5 mM strontium. Day 8 of control static condition corresponds to the D8 CTL data. Error *bars* represent S.E.M. *p < 0.05 and **p < 0.01 compared to media without strontium (*N* = 3 independent experiments)

Unlike the protein assay, VEGF 120 expression (soluble form), varied little over time and with the strontium treatment (Fig. 6a). Concerning VEGF 164, changes under strontium were not significant, except for a slight tendency to increase when treated with strontium during osteogenesis (Fig. 6b). As expected, the VEGF 188 isoform expression similarly matched the protein assay on cell lysates, indicating that VEGF188 isoform is probably the main form of the cell-associated VEGF (Fig. 6c). Under our conditions, AD is characterized by a late upregulation of VEGF164 and 188 (not 120); whereas strontium downregulates them (−35% and −67 %, respectively). Interestingly, under osteogenic conditions we observed a significant increase in VEGF188 isoform expression under strontium at day 8 (+58 %, *p* < 0.001). Static controls showed a comparable profile for VEGF 188 isoform in AD and OB conditions. VEGF 164 and 120 were different, being significantly inhibited by rotation in AD and OB + strontium condition (−54%, −62%, −55% and −73 %, respectively, *p* < 0.05 to *p* < 0.001). In summary, we established that mostly VEGF188 was differentially affected by strontium in our conditions, VEGF188/VEGFc expression being increased in osteogenic conditions and repressed in adipogenic conditions. Nevertheless, in uncommited cells, we were not able to establish a link between the observed release of VEGF (from D2 to D6; Fig. 4) and VEGF isoforms expressions.

### Soluble or cell-associated VEGF differentially regulates RhoGTPases activities

**Fig. 7:**
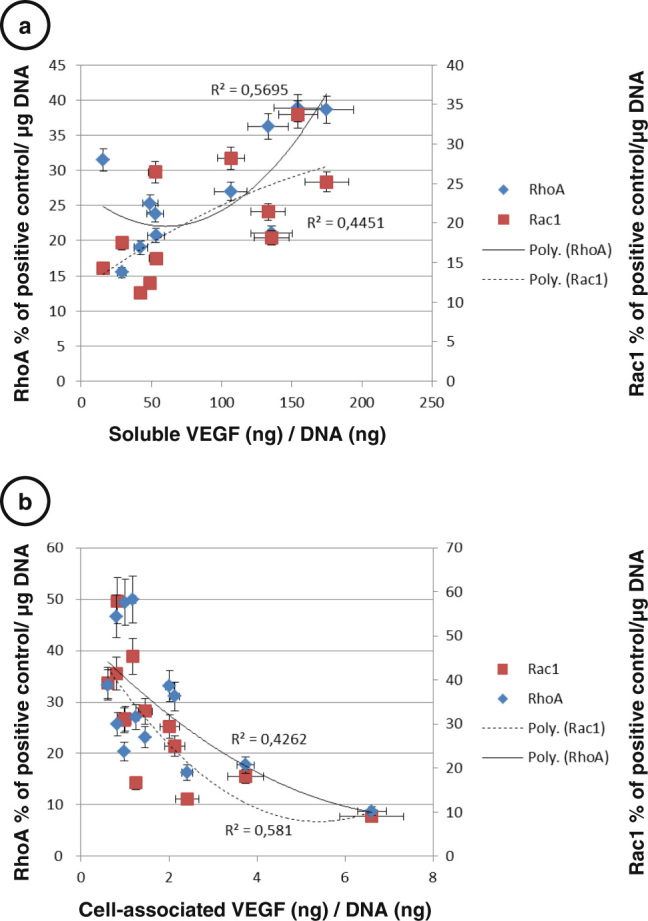
Spearman’s rank correlations with significant associations for VEGF and RhoA and Rac1 activities**. a** Soluble VEGF secretion was associated with increased RhoA and Rac1 activities, *p* < 0.05. **b** Cell-associated VEGF (trapped VEGF) was negatively associated with RhoA and Rac1 activities, *p* < 0.05. Error *bars* represent S.E.M. (*N* = 3 independent experiments)

In order to understand the influence of soluble vs. cell-associated VEGF, we correlated ELISA measurements of VEGF with RhoA and Rac1 activities (Fig. 7). We chose to merge all the different conditions because we were interested by the general effect of soluble or cell-associated VEGF on RhoGTPase. Furthermore, the effect of strontium on commitment of C3H10T1/2cells is the opposite in OB and AD conditions; thus comparison between committed or uncommitted cells was not possible without merging strontium-treated cells and not treated ones. In these conditions, VEGFs secretion was found to be associated with increased RhoA and Rac1 activities (Fig. 7a, *p* < 0.05), whereas VEGFc (trapped VEGF) correlated negatively with RhoA and Rac1 activities (Fig. 7b, *p* < 0.05). These correlations highlight that RhoA and Rac1 activation or inhibition are always parallel and that the role of VEGF on RhoGTPases is complex and dependent on VEGF location (soluble or trapped into matrix or cell).

## Discussion

The first goal of this study was to investigate the effect of strontium chloride on the balance between OB and AD of multipotent cells under simulated microgravity condition. The originality of this work resides in the comparison of AD and OB in parallel experiments using the same cell model. During OB, we confirmed using C3H10T1/2 an alteration of the osteogenic commitment, with osteogenic markers remaining particularly stable as compared to 3D static controls; hence indicating that OB was not stimulated but limited during microgravity condition, in accordance to previous reports.^32,33^ AD was found to be stimulated by rotation as compared to static controls, indicating that simulated gravity accelerates commitment of multipotent cells toward adipocytes as already documented.^36^ Addition of strontium chloride allowed an upregulated commitment in osteoblasts throughout the time of culture. In parallel, we clearly established in AD condition that strontium was able to inhibit AD as already demonstrated in conventional 2D cultures,^31,37^ but never in altered gravity conditions. Our results indicate thus that strontium is a particularly good counter measure for space-related activities using multipotent cells as it can limit AD and promote OB. More interestingly, we observed that strontium effects are identical in different mechanical contexts and culture models, demonstrating that strontium acted independently of the mechanical context of the multipotent cells.

In an attempt to explain such ability, we tested the role of strontium on RhoGTPases. Many authors have reported that the reduced osteoblastic differentiation observed during culture under simulated microgravity and spaceflight^14,16,38–41^ could be explained by the cytoskeletal disruption occuring in MSC, accompanied by an actin collapse and a loss of focal adhesion and stress fibers.^15^ This disruption of the actin cytoskeleton could be attributable to a reduced RhoA activity under microgravity conditions.^5,19^ Nevertheless, whereas AD is characterized by a decrease in RhoA activity and a Rac1 activity that remained low in microgravity condition, confined OB did not show a lowering of RhoA or Rac1 activity. Strontium-treated cells in OB and AD conditions highlighted an upregulation of both active forms of RhoA-GTP and Rac1-GTP as compared to untreated cells. The ability of strontium to stimulate OB and inhibit AD of C3H10T1/2 cells was in accordance with many previous reports,^30,42^ but the activation of the both RhoGTPases has never been reported.

The most important information provided in this paper is that activation of both RhoA and Rac1 was associated with OB. Concerning RhoA activity, it was demonstrated that RhoA activation is mandatory for OB^43,44^ and that RhoA activation is responsible for fibrillogenesis of fibronectin triggering OB in mesenchymal cells.^45^ For Rac1 activity, it has been established that its activation is associated with the production of reactive oxygen species (ROS),since Rac1 is a regulatory unit of NADPH oxidase. An increase in ROS is commonly linked with AD promotion, but ROS also act as signaling regulators for OB activation. Moreover, Cristancho *et al.* ^46^ showed that Rac1 needed to be reduced to allow adipocyte maturation. Activation of Rac1 in strontium-treated cells could be sufficient to block AD. Rac1 is also known to control β-catenin nuclear translocation, regulating shear stress-driven β-catenin signaling in osteoblasts^18^ and is required for OB and bone development.^17^ In contrast to the literature that opposes RhoA (cell contractility) and Rac1 (cell relaxation) activities, we provide evidence that strontium is able to activate both. The dual activation of RhoA (for fibrillogenesis) and Rac1 for β-catenin pathway may reveal a new way to control OB and limit AD, particularly in situations where OB is impaired, such as under microgravity conditions.

In this context, VEGF signaling is the first target to explain RhoA and Rac1 activations through a conventional VEGF/VEGFR/RhoGTPase pathway.^47,48^In uncommitted cells (D2-D6), secretion of VEGF is associated with RhoA and to a lesser extend with Rac1 induction when cell are treated with strontium, whatever the differentiation context. We were not able to completely explain the release of VEGF by upregulation of a particular VEGF isoforms. This discrepancy leads us to propose that VEGF signaling is not directly linked to transcription of VEGF but probably also to its location (trapped into matrix or soluble). VEGF signaling could be needed for OB induction as already mentioned by many authors.^21–23^ The pioneering work of Carmeliet’s group on VEGF signaling in bone established that VEGF164 can stimulate β-catenin pathways known to support OB^49^ and Pierre Marie’s observations already showed that strontium acts through a RhoA-dependent process to stimulate Wnt signaling.^30^

Concerning AD, we observed that VEGFc expression was dramatically upregulated under AD conditions and repressed upon strontium treatment. Hovey et al*.* ^50^ showed indeed that VEGF188 (matrix-bound VEGF) is rather found in differentiation of terminal adipocytes, unlike VEGF 120 and 164, which are expressed at earlier stages. Inhibition of VEGF188 found in our strontium-treated cells could limit AD by blocking terminal differentiation.

In our hands, expression of VEGF receptors were found to be particularly low, precluding satisfactory explanations concerning strontium effects and further VEGFR signaling hypothesis. Interestingly, the positive association of VEGFs with RhoA and Rac1 activations might explain for uncommitted cells that OB is supported^51^ but AD repressed. Further studies will be necessary to identify which VEGF receptors are involved in this association. On the contrary, after cell commitment in OB condition, immobilization of VEGF into matrix reduced the amount of soluble form; limiting RhoA and Rac1 activations (D8). RhoA activation in uncommitted cells and inhibition for committed cells has been already proposed by Irie et al*.* ^52^ Our results are pointing out that both RhoA and Rac1 might be following such an activation profile. Concerning VEGFc (matrix-bound or intracellular VEGF), the negative association between VEGFc and RhoGTPases nicely explains why RhoGTPases are inhibited after commitment. In AD conditions, it seems that in the absence of a significant amount of matrix to trap VEGF, strontium activates the release of VEGF (solubilization), thus limiting negative effects on RhoA and Rac1 of VEGFc. This phenomenon even more sustains the positive effects of VEGFs, explaining the constant increase of RhoGTPases under strontium in AD conditions, whatever the differentiation status and their inability to maturate into adipocytes.

All together, we provide evidence that strontium is particularly efficient for conteracting AD and activating OB in a multipotent cell model, even cultivated in a simulated microgravity environment. Our data support the hypothesis that strontium acts in uncommitted cells by activation of both RhoA and Rac1 GTPases. This dual activation might be able to explain many of the described effects of strontium *in vitro,* in particular its ability to sustain Wnt/β-catenin signaling already known to be crucial in OB promotion and AD limitation. We also pointed out that strontium effects might be mediated through VEGF location (cell-associated or soluble). Manipulation of VEGF splicing (mechanical stress, pH and progesterone) might be necessary to establish that a unique VEGFc signaling dictates stem cell fate.

## Materials and methods

### Reagents

Cytodex 3, Minimum Essential Medium Eagle—Alpha Modification (αMEM), Dulbecco’s modified Eagle medium (DMEM), l-ascorbic acid, β-glycerophosphate, retinoic acid, strontium chloride, oil red O, trypsin-EDTA, phosphate-buffered saline (PBS), glycerol phosphate calcium salt, phosvitin from egg yolk, phosphatase alkaline from bovine intestinal mucose, tris(hydroxymethyl)amino-methane, polyethylene-sorbitan monolaurate (Tween), Triton X-100 and TRI-Reagent were purchased from Sigma Aldrich (St-Louis, USA). Rosiglitazone was obtained from Interchim (Montluçon, France). RiboGreen RNA and PicoGreen DNA quantitation kits were purchased from Invitrogen (Life Technologies, Eugene, OR, USA). RNeasyPlus Mini Kit was purchased from Qiagen (Courtaboeuf, France). Light cycler-FastStart DNA Master, SYBR Green I dye (Lightcycler faststart DNA master SYBR Green I) was obtained from Roche Diagnostics (Meylan, France). Protein assay kit Pierce® BCA (bicinchoninic acid) was obtained from Thermo Scientific (Rockford, USA). Nonidet P-40 substitute was purchased from Fluka Biochemika (Steinheim, Germany). Fetal bovine serum (FBS) was acquired from Biological Industries (Kibbutz Beit Haemek, Israel).

### RWV bioreactor

The NASA RWV bioreactor (size 120 mL) was purchased from Synthecon (Houston, USA). It consists of a cylindrical growth chamber that contains a flat silicone rubber gas transfer membrane for oxygenation. The fluid dynamic principles of the RWV bioreactor allow oxygenation without turbulence, leading to a reduced fluid-shear force of less than 0.001 Pa^53,54^ and a theoritical average g-vector of 10^−2^–10^−3^.^55^ The culture technique commonly employed is to first inoculate cells on microcarrier beads, and then to culture them in the rotary vessel.^56^ For the cell seeding, the ratio 10 mg beads/10^6^cells was used. Speeds were adjusted according to the level of aggregation of our culture: 60–80 rpm for adipocytes cultures and 75–95 rpm for osteoblasts cultures, as the latter presented larger aggregates (data not shown).

### Beads mineralization

Beads with apatite collagen complexes (ACCs) were prepared using a previously described method^57^ with slight modifications. Briefly, Cytodex 3 microcarriers already coated with collagen were placed in 35 mm cell culture-treated dishes, and immersed in various baths based on Tris-buffered saline at 200 mM, pH = 8.5. The first bath contained alkaline phosphatase and egg yolk phosvitin (0.4 mg/ml) at 37 °C, and the second contained calcium β-glycerophosphate (6 mM) at 37 °C. These successive immersions induced the deposition of a hydroxyapatite-mimicking bone mineral on the beads.^57,58^ The duration and number of successive incubations determined the amount of apatite mixed with the collagen. A 3-–10-h (first bath-second bath) cycle provided an ACC where 20 % of the collagen surface was mixed with apatite. This cycle was repeated twice and thus led to an ACC where 40 % of the collagen surface was mixed with apatite, which was used in the experiments for osteogenic differentiation. The beads were finally washed with water followed by αMEM before cells seeding.

### Cell culture

The mouse multipotent mesenchymal cell line C3H10T1/2 (clone-8; American Type Culture Collection, LGC Promochem, Molsheim, France) was maintained in T75 flasks in DMEM supplemented with 10 % FBS, 2 mM -l-glutamine, and antibiotics (50 U/ml penicillin and 50 μg/ml streptomycin), in a humidified atmosphere of 5 % CO_2_ at 37 °C. In order to avoid any bias in comparing the different conditions, the same C3H10T1/2 cells were used at the same duration of differentiation for AD and OB. In these experiments, cells were used at passage 12 and a new ampoule of cells from liquid nitrogen storage was thawed before each experiment. In all, 20.10^6^ cells were seeded on 200 mg of microcarrier beads either mineralized or not, in 5 ml αMEM during 4 h at 37 °C and then cultured in 90 mm Petri dishes untreated for culture with 10 ml of medium. After 2 days of preculture in this proliferation media, C3H10T1/2 cells were differentiated in an adipogenic or osteogenic medium. The AD medium consisted of complete αMEM with 1 µM of rosiglitazone, a potent agonist of PPARγ2, and was used with plastic beads. The OB medium comprised 5 mg/mL of l-ascorbic acid, β-glycerophosphate at 10^−3^ M and retinoic acid at 10^−5^ M, and was used with mineralized plastic beads. The AD or OB media were renewed every 2 days. The cells were treated with 5 mM of strontium at days 0 and 2. Strontium chloride powder was diluted in sterile water and then filtered. For the experiments, it was used at 5 mM and was added at each medium renewal. When added in the RWV, the strontium chloride half-life (several years^59^) allows it to last until the end of the experiment, 6 days later, and a total renewal of the 120 mL of the RWV medium could thus be avoided. For each analysis day, a sample of beads was extracted from the bioreactor or the Petri dishes and divided in order to perform staining, RNA, proteins or RhoGTPases activities measurements. Each experimental conditions were repeated three times independently (four times for RhoGTPases analysis).

### RNA extraction and RT-PCR

RNA extraction and RT-PCR were performed on C3H10T1/2 cells to analyze cell differentiation. C3H10T1/2 cells seeded on beads were harvested after 2 days of preculture, and after 2, 6 and 8 days in AD or OB culture media (Fig. 1). Total RNA was isolated using TRI-Reagent according to the manufacturer’s instructions. Briefly, chloroform was added to separate the aqueous phase containing RNA from the interphase and the organic phase. The aqueous phase was recovered and precipitated with isopropanol. The RNA pellets were then washed in 70 % ethanol. Finally, after air-drying for 1–2 min, RNA pellets were dissolved in RNase-free water and then purified with the RNeasyPlus Mini Kit. RNA concentration was assessed with the Ribogreen kit. RNA quality was checked with the Experion automated electrophoresis station (BIO-RAD, Hercules, CA, USA). Complementary DNA (cDNA) was synthesized from 2 µg of total RNA with the iScriptTMcDNA Synthesis kit for Thermocycler (BIO-RAD, Hercules, CA, USA) according to the manufacturer’s instruction. For quantitative RT-PCR, 0.08 µg of cDNA mixture was prepared for a CFX96TM (BIO-RAD, Hercules, CA, USA) RT-PCR detection system, using SYBR Green I dye (Lightcyclerfaststart DNA master SYBR Green I). Primer sequences of the markers are listed in Table 1. For each gene, expression levels were normalized to β-actin expression, which did not change during the culture time and the conditions used (not shown). Amplified products were examined for size estimation on a 2 % agarose gel with 1 μg/ml ethidium bromide and a DNA molecular weight marker.

**Table 1:** Primer sequences

Genes	Forward	Reverse
**β-actin** NM_007393.3	cctctatgccaacacagtgc	tctgctggaaggtggacagt
**PPARγ2**NM_001127330.1	tccgtgatggaagaccactc	ccacagactcggcactcaat
**FABP4** NM_024406	ggaacctggaagcttgtctc	ttcctgtcgtctgcggtgat
**C/EBPα** NM_007678.3	caagccaggactaggagatt	ccaaggcacaaggttacttc
**Adiponectin** NM_009605	gtgatggcagagatggcact	cacataagcggcttctccag
**ALP (Akp2)** NM_007431.2	agttactggcgacagcaagc	ggacctgagcgttggtgtta
**OSX (Sp7)** NM_130458.3	atggcgtcctctctgcttg	aaggtcagcgtatggcttct
**BSP** NM_008318.3	acaatccgtgccactcactc	ggaactatcgccgtctccat
**OPN** NM_009263.2	cccggtgaaagtgactgattc	atggctttcattggaattgc
**Flt-1** NM_010228.3	gcacattggtggtggctgac	ctctccttcggctggcatct
**KDR** NM_010612.2	gcggagacgctcttcataat	cacttgctggcatcataagg
**VEGF 188**	catgcggatcaaacctca	ctccaggatttaaaccggga
**VEGF 164**	catgcggatcaaacct	cacagtgattttctggct
**VEGF 120**	catgcggatcaaacct	gtcacatttttctggct
**Nrp-1** NM_008737.2	agcaagcgcaaggctaagtc	tggtcgtcgtcacactcatc

*PPAR* peroxisome proliferator-activated receptor, *ALP* alkaline phosphatase, *OSX* osterix, *BSP* bone sialoprotein, *OPN* osteopontin, *VEGF* vascular endothelial growth factor

### DNA measurements

The PicoGreen DNA quantitation kit was used to measure double-stranded DNA concentrations in solution. All reagents (dsDNA reagent, Tris-EDTA buffer: 200 mM Tris—HCl, 20 mM EDTA, pH 7.5 and lambda DNA standard) were obtained from the kit and the assay was performed as outlined in the protocol from the manufacturer. The data were corrected for cell-free values. Samples were placed in a black 96-well plate and excited at 485 nm. The emission was measured at 538 nm using a fluorometer (CFX96TM, BIO-RAD, Hercules, CA, USA).

### RhoA and Rac1 G-LISA assays

Levels of active forms GTP-RhoA and GTP-Rac1 were analyzed by assessing RhoGTPases activities using respective G-LISA^TM^ assays [(RhoA, Rac1 small GTPases-linked immunosorbent activation assay (G-LISA); colorimetric format; Cytoskeleton from Tebu-Bio (Le Perray en Yvelines, France))] following the manufacturer’s protocol. A positive control included in the kit allowed comparison between the different measurements of several experiments by always adding the same active RhoGTPase protein amounts in the control wells (0.24 µg). It corresponds to RhoA or Rac1 control proteins (lyophilized constitutively active RhoA or Rac1). Values obtained during experiments were normalized to the positive control and then to the DNA content of the cell lysates, measured with a PicoGreen DNA quantitation kit.

### Vascular endothelial growth factor enzyme-linked immunosorbent assay

VEGF content was measured in sample lysates (matrix-bound or intracellular VEGF) and media (soluble VEGF) with an ELISA kit (Mouse VEGF DuoSet, R&D, France), according to the manufacturer’s instructions. Briefly, samples were lysed in lysis buffer composed of PBS with 0.5 % Nonidet P-40, and 1 % proteases inhibition cocktail. Each sample was run in duplicate in the assay and fluorescence was determined with a multiwell plate spectrophotometer (Multiskan spectrum microplate spectrophotometer). The data were corrected for alone lysis buffer values, determined from a standard curve and normalized to the DNA content of the cell lysate, measured with a PicoGreen DNA quantitation kit. Concerning media for soluble VEGF, media of samples were run without lysis and data were corrected with alone media values and normalized to the total protein content of the media, measured by the Protein assay kit Pierce® BCA (bicinchoninic acid).

### Statistical analysis

The obtained data were analyzed with the GraphPad Prism 5 and GraphPadInstat 3 softwares. Each condition group followed the Kolmogorov-Smirnov normality test and showed similar variances. The results from each group were compared using two-ways analysis of variance, followed by Bonferroni Multiple Comparisons post hoc test. A *p*-value <0.05 was considered statistically significant. Concerning Fig. 7, Spearman’s rank correlations with significant associations for RhoGTPases activity and ELISA measurements of VEGF were presented for *p* < 0.05.
